# Lifespans of Twins: Does Zygosity Matter?

**DOI:** 10.3390/genes10020166

**Published:** 2019-02-20

**Authors:** Jacob Hjelmborg, Pia Larsen, Jaakko Kaprio, Matt McGue, Thomas Scheike, Philip Hougaard, Kaare Christensen

**Affiliations:** 1 Department of Epidemiology and Biostatistics, University of Southern Denmark, DK-5000 Odense, Denmark; plarsen@health.sdu.dk (P.L.); mcgue001@umn.edu (M.M.); phou@lundbeck.com (P.H.); kchristensen@health.sdu.dk (K.C.); 2 The Danish Twin Registry, University of Southern Denmark, DK-5000 Odense, Denmark; 3 Department of Public Health, University of Helsinki, FI-00014 Helsinki, Finland; jaakko.kaprio@helsinki.fi; 4 Institute for Molecular Medicine FIMM, University of Helsinki, FI-00014 Helsinki, Finland; 5 Department of Psychology, University of Minnesota, Minneapolis, MN 55455, USA; 6 Department of Biostatistics, University of Copenhagen, DK-1014 Copenhagen K, Denmark; bhd252@ku.dk; 7 Biometric Division, Lundbeck, DK-2500 Valby, Denmark

**Keywords:** lifespan, mortality, twins, zygosity, unknown zygosity, cumulative incidence curves, age-stratification, long-livers

## Abstract

Studies with twins provide fundamental insights to lifespans of humans. We aim to clarify if monozygotic and dizygotic twin individuals differ in lifespan, that is, if zygosity matters. We investigate whether a possible difference in mortality after infancy between zygosities is stable in different age cohorts, and whether the difference remains when twins with unknown zygosity are taken into account. Further, we compare the distribution of long-livers, that is, the upper-tail of the lifespan distribution, between monozygotic and same-sex dizygotic twin individuals. The Danish Twin Registry provides a nationwide cohort of 109,303 twins born during 1870 to 1990 with valid vital status. Standard survival analysis is used to compare mortality in monozygotic and dizygotic twin individuals and twin individuals with unknown zygosity. The mortality of monozygotic and dizygotic twin individuals differs slightly after taking into consideration effects of birth- and age-cohorts, gender differences, and that twins are paired. However, no substantial nor systematic differences remain when taking twins with unknown zygosity into account. Further, the distribution of long-livers is very similar by zygosity, suggesting the same mortality process. The population-based and oldest twin cohort ever studied suggests that monozygotic and dizygotic twins have similar lifespans.

## 1. Introduction

One of the advantages of using twins in quantitative genetic modelling is the assumption of comparability (of trait means and distribution of variances) of the monozygotic (MZ) and dizygotic (DZ) twins with respect to the studied trait or disease when considering the twins as individuals [1]. However, for studies on disease onset and other age-dependent traits, an important factor to study is the relative survival of MZ twins compared to DZ twins [2]. One may speculate whether MZ twins in general show better survival in comparison to same-sex and opposite-sex DZ twins as an effect of greater closeness and social support. On the other hand, MZ twins show lower birth weights, higher risk of prematurity and greater neonatal mortality than DZ twins, which may affect long term survival [3]. Genetic and environmental effects on mortality are well studied [4,5], showing a modestly higher concordance of overall mortality in MZ twin pairs than DZ twin pairs. However, the effect of zygosity on concordance in mortality might integrate social factors beneficial for a longer life. It may be difficult to disentangle such factors, and many studies points towards little or no difference in twins as individuals across zygosity for many outcomes related to survival [6,7].

Compared to the background population, twin individuals have higher perinatal and infant mortality [8], however the relatively high mortality in twins occurs soon after birth [2], and after the age of six mortality of twins is similar to that of the background population [6]. A recent study on Danish twin birth cohorts from 1870–1900, left truncated at age 10, suggested that MZ twin individuals have better survival than same-sex DZ twin individuals at nearly all ages [9]. The paper applied a two-process model, assuming independence between all subjects, and partitioned mortality into an intrinsic process, where death follows from cumulative and incremental degradation of survival capacity, and an extrinsic process, where death results from an acute environmental challenge [9]. No formal statistical tests were made, but explorative results indicated that MZ female twins may have better overall survival than same-sex DZ female twins, due to greater extrinsic survival. Monozygotic male twins may have better overall survival than same-sex DZ male twins, where the improved survival was ascribed to greater extrinsic survival until age 65–70, and to greater intrinsic survival from age 65–70. Based on these results, we seek here to study rigorously mortality after infancy in MZ, same-sex DZ, and opposite-sex DZ twin individuals, accounting for the dependency within twin pairs, in birth cohorts ranging from 1870 until 1990 and using the most recent follow-up data on vital status in the Danish Twin Registry [10,11].

The Danish Twin Registry was established in 1954, identifying twin pairs prospectively as well as retrospectively from church records and classifying zygosity through questionnaires and interviews with surviving twins and family [11,12]. The register includes Danish twin pairs from birth cohorts 1870 onwards and is regarded almost complete from birth cohort 1960 [11,13]. For some same-sex twin pairs the zygosity is not determined, in particular in the early birth cohorts and among twin pairs with early mortality. In the early birth cohorts from 1870 to 1960, more same sex twins with early mortality are likely to be registered as unknown zygosity (UZ) due to the ascertainment method, and consequently, mortality of UZ twins from birth cohorts 1870–1960 may be expected to be higher than for twins registered as MZ or DZ. From birth cohorts 1960, it may be assumed that the registration of UZ twins due to death after infancy is negligible [10]. Although the true zygosity of UZ twins is unknown, it is expected that UZ twin pairs are a mixture of MZ and same-sex DZ twin pairs. 

The objectives of this study are to compare mortality after infancy in MZ, same-sex DZ, and opposite-sex DZ twin individuals, to examine whether possible differences in survival between zygosities are stable in different and age cohorts, and to investigate differences in mortality in MZ, same-sex DZ, and opposite-sex DZ under different assumptions on the true zygosity of UZ twin pairs. A further objective was to explore the upper-end of the lifespan distribution in MZ and same-sex DZ twin individuals.

## 2. Materials and Methods 

### 2.1. The Danish Twin Registry

As the first nationwide twin registry, the Danish Twin Registry was established in 1954, identifying twin pairs prospectively as well as retrospectively from church records of all 2200 Danish parishes and all calendar years 1870–1930. Also, regional population registers and other public sources were used to identify twins and close relatives of twins. All twins, or their closest relatives, were sent questionnaires, including questions on similarity between the twins to determine the zygosity of same-sex twin pairs [6,11,12,14,15]. If one or both twins had died or emigrated before the age of six, the twin pairs were not followed up. Twins, who did not reply to the questionnaires, as well as a minority providing inconsistent responses, were classified as UZ [10]. Later comparisons of the zygosities of same-sex twins determined from the questionnaires with results determined from blood samples indicated that more than 95% of twin pairs were classified correctly [16].

For the birth cohorts 1930–1990, the ascertainment procedure was based on the civil registration system introduced in 1968 and hence the criterion for inclusion was survival of both twins until 2 April 1968, which resulted in complete registration of all twins born from that date [10].

### 2.2. Study Population

**Figure 1:**
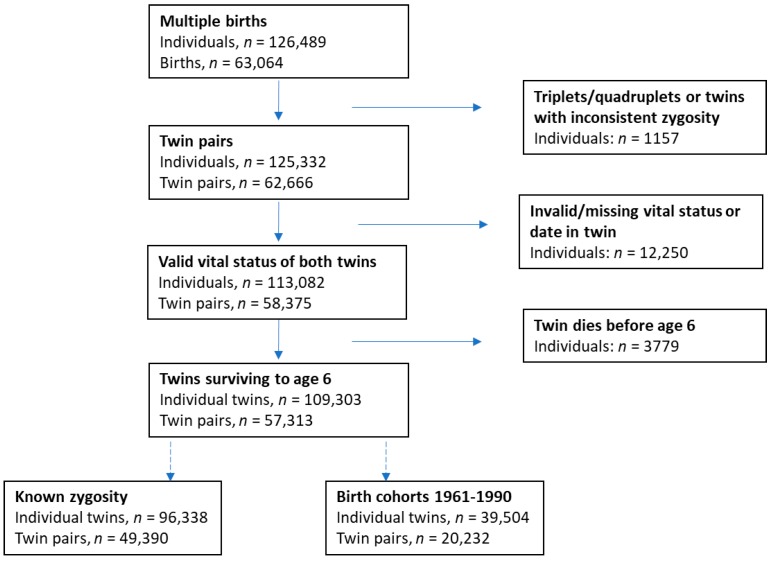
Flowchart.

The study includes population-based birth cohorts from the Danish Twin Registry including information about mortality. The Danish Twin Register comprises total of 126,489 individuals from multiple births from birth cohorts 1870–1990. Excluding triplets, quadruplets, twin pairs with inconsistent zygosities, twin individuals with invalid or missing vital status, and twin individuals who died before age six, leaves a total of 109,303 twin individuals from 57,313 twin pairs. For our primary analyses on differences in mortality between zygosities, we use all twin individuals with known zygosity from birth cohorts 1870–1990 (*n* = 96,338), and for our secondary analyses we include UZ twins and restrict the birth cohorts to 1961–1990 (*n* = 39,504) where there is virtually complete vital status follow-up of the twin individuals [10] (Figure 1). 

The project was approved, and data were provided by, the Danish Twin Registry (ref. no. 17/64840) in an anonymized fashion. Access to the data requires application to this registry. According to Danish law, no ethical approval is required for registry-based studies.

### 2.3. Statistical Analyses

Individual twins were excluded in case of missing data on vital status or date of vital status, or in case of death before age six years old (Figure 1). For the general population, it is well known that life expectancy has improved over the period 1870–1990. To be able to handle this period-effect and the differences in ascertainment methods, the birth cohorts are divided into four cohorts within which the same ascertainment method was used, and within which mortality rates are assumed to be homogeneous in the background population: In the first two cohorts, church records were used for identification of twins; this cohort was further divided into two separate cohorts, due to the pronounced increase in life expectancy in the Danish background population during this period, increasing from an average life expectancy in males between 46–49 years in 1870–1900 to an average life expectancy in males between 53–61 years in 1901–1930 (StatBank Denmark, Statistics Denmark). In the third cohort, including birth cohorts 1931–1960, the ascertainment of twins was based on survival of both twins until April 1968 and the average life expectancy in the background male population ranged between 62–70 years. From 1960, virtually all twins surviving infancy were included, and the average life expectancy in the background male population was between 70–72. Descriptive analyses were conducted for each cohort separately. Mortality was compared between zygosities (MZ: monozygotic, SSDZ: same-sex dizygotic, OSDZ: opposite-sex dizygotic) separately within each cohort using Kaplan-Meier plots and cumulative incidence curves [17] of death by age, stratified on sex. Due to non-proportional hazards and statistically significant interaction between zygosity and age, the models were stratified on three age-intervals (0–50 years, 50–75 years and 75+ years) by introducing a time-varying interaction term with cut-points at ages 50 years and 75 years. Within each age-interval, the proportional hazards assumptions were satisfied when assessed using Schoenfeld residual tests. To combine survival analyses across the four cohorts, the baseline hazards were stratified on the cohorts in the age-stratified Cox proportional hazard models. In analyses on both sexes combined, the baseline hazards were further stratified on sex. The mortality analyses were conducted on twins regarded as individuals. However, as the two twins within a twin pair are not independent, cluster robust standard errors were used in the statistical models to account for the lack of independence between observations [18]. All analyses were conducted on both sexes combined and for each sex separately. 

Registration of twins surviving infancy is assumed to be complete in the Danish Twin register from 1960 onwards. Therefore, the fourth cohort (birth cohorts 1961–1990) was used in three sensitivity analyses including all twin individuals with known or unknown zygosities. The cohort comprises a total of 39,504 twin individuals of which 5178 have UZ. In the first sensitivity analysis, all UZ twin pairs were treated as MZ, in the second UZ twin pairs were randomized 1:1 as either MZ or SSDZ twins, and in the third, UZ twin pairs were randomized 1:2 as either MZ or SSDZ twins. In the cohort 1961–1990, the follow-up time was at most 56 years (from 1 January 1960 until end of follow-up 1 October 2016), and most twin individuals were alive at the time of follow-up. Age-stratified (0–50 years and 50+ years) Cox proportional hazard regression analyses with cluster robust standard errors were conducted. In analyses on both sexes combined, the baseline hazards were stratified on sex. The analyses were conducted on both sexes combined and for each sex separately. Assumptions on proportional hazards were assessed using Schoenfeld residual tests and were satisfied for both sexes and in both age-groups.

Finally, we explored whether the distribution of the lifespans of long-livers, that is, twin individuals reaching very high ages, differ between MZ and SSDZ twin individuals, by comparing the upper-tail of the lifespan distributions. To do this, the generalized extreme value distribution (GEV) was applied to model the upper-tail of the lifespan distribution for twins born between 1870–1930 [19]. The GEV is a three-parameter model with a location parameter µ, estimating the center of the lifespan distribution for twin individuals, a scale parameter σ, estimating the deviations around the location parameter, and finally a shape parameter ξ, estimating the heaviness of the upper-tail lifespan, i.e., the distribution of lifespan among the long-livers: twins reaching very high ages. An increasing shape parameter corresponds to a higher probability of reaching very long lifespans. A negative value of the shape parameter indicates a light upper-tail, i.e., that there is a final limit to the highest possible lifespan, which is in accordance with human lifespans [20]. The shape parameter of the GEV to be reported for MZ and SSDZ for each gender was estimated by the moments method and inference was obtained by parametric bootstrap using the R-package ExtRemes [21].

## 3. Results

Characteristics of twin individuals with known zygosities in each of the four cohorts are shown in Table 1. The age at follow-up (FU) was lower in the third and fourth cohorts due to censoring at end of the study period (1 October 2016). In the third cohort, the proportions of MZ twins and females were lower than in the other cohorts. In the second cohort, the proportion of OSDZ twins was lower than in the other cohorts.

Kaplan-Meier survival curves indicate non-proportional hazards in the two first cohorts for both male and female twins (Supplementary Figures S1 and S2). For younger ages, the mortality of OSDZ twin individuals appears much higher than both MZ and SSDZ twin individuals in the first two cohorts, while at higher ages the survival is similar for all three zygosities. 

In all three age-intervals, OSDZ twin individuals have significantly higher mortality than MZ twin individuals for both males and females (Table 2). For males, SSDZ twin individuals have significantly higher mortality than MZ twin individuals in all three age-intervals, and for females SSDZ twin individuals have significantly higher mortality than MZ twin individuals in the two younger age intervals. For both males and females, the difference in mortality between MZ and DZ twin individuals is largest in the youngest age interval (0–50 years) and weakens with age (Table 2). 

Secondary analyses were conducted on the birth cohorts 1961–1990 including UZ twin pairs. Population characteristics of the twin individuals are shown in Table 3. 

Kaplan-Meier survival curves (Figure S3) suggest a higher mortality in UZ twin individuals than twin individuals with known zygosity, mainly among male twins. Age stratified analyses on the birth cohorts 1961–1990 in Table 4 show that the increased mortality among UZ male twin individuals mainly exists in the younger age group (up to 50 years of age), while there are no statistically significant differences in mortality in twin individuals aged above 50. 

There are no interactions between zygosity and age-group among male or female twins (all: *p* = 0.696, males: *p* = 0.519, females: *p* = 0.902). In all three sensitivity analyses, treating all UZ twin pairs as MZ, randomizing UZ twin pairs 1:1 as MZ or SSDZ twin pairs, or randomizing UZ twin pairs 1:2 as MZ or ss-DZ twin pairs, there are no differences in mortality between MZ and DZ twin individuals in neither age group (Supplementary Tables S1–S3).

For MZ and SSDZ twin individuals, the upper-tail of lifespan distributions are similar for both male and female twins. Overall for the birth cohorts 1870–1930, the estimated values of the shape parameters, reflecting the probability of reaching very long lifespans, are very similar for MZ males and DZ males (MZ: ξ (95%-CI) = −0.74 (−0.76, −0.69); DZ: ξ (95%-CI) = −0.75 (−0.77, −0.72)), and for MZ and DZ females (MZ: ξ (95%-CI) = −0.86 (−0.88, −0.81); DZ: ξ (95%-CI) = −0.84 (−0.86, −0.81)). Hence, the shape of the upper-tail of lifespans are very similar by zygosity suggesting the same mortality process regarding the probability of reaching very long lifespans. 

## 4. Discussion

### 4.1. Main Findings 

Using mortality information for the 1870–1990 Danish twin cohorts, we find that the mortality after infancy of MZ and DZ twin individuals differ slightly after taking age, sex, and birth cohort into account. However, when further taking into consideration that some twin pairs have unknown zygosity, we find no indication of a substantial or systematic difference in survival between MZ and DZ twin individuals. Sensitivity analyses, treating UZ twin pairs as MZ twins or randomizing UZ twin pairs as either MZ or DZ twins, indicate that any apparent differences in mortality after infancy between MZ and DZ twin individuals disappear when accounting for UZ twins, suggesting that the differences in mortality between MZ and DZ twins may be explained by selection due to unknown zygosity of some twins. Finally, we find that the distribution of long-livers, that is, the upper-tail of the lifespan distribution, is very similar in MZ and SSDZ twin individuals suggesting the same mortality process regarding the probability of reaching very long lifespans. 

### 4.2. Comparisons with Other Studies

In line with the results of Sharrow and Anderson [9], survival analyses, not accounting for UZ twins, suggest better survival of MZ than DZ twin individuals in the younger age groups. However, these results might reflect a deselection of UZ twins with higher mortality than twins with known zygosity. The differences in mortality after infancy between MZ and DZ twin individuals vanish when UZ twins are merged with twins with known zygosities under different conditions. The analyses in the present paper as well as the paper by Sharrow & Anderson [9] are based on left truncated data, conditioning on both twins surviving to age six (present paper) or 10 (Sharrow and Anderson [9]), and may thus include only the most robust twin pairs where both of them were strong enough to survive their youngest childhood. Including the individual surviving twin from all pairs in which one twin died under the age of six would enhance the comparability with the general population on mortality after infancy. Unfortunately, there are no data to enable follow-up of such surviving twins from the early cohorts. 

That MZ and DZ twins derive from the same base population with respect to lifespan is consistent with other literature indicating no or little differences between MZ and DZ twin individuals, and between twins and singletons in adulthood with respect to mortality, lifestyle, and incidence of common diseases [6,22,23,24,25,26]. Data from multiple twin cohorts around the world have formed a substantial part of genome-wide association studies of common traits and complex diseases and there is no evidence to show that the gene–disease associations seen in singletons and twins differ. Thus, being a twin does not appear to impact the basic biological processes and human development in adolescence and adulthood. This implies that findings from twins are generalizable to the population as a whole. Given that twin studies often have response rates higher than surveys in the population at large, twin studies can be considered more representative when based on large, population-derived twin cohorts such as the Danish Twin Registry and other Nordic cohorts.

### 4.3. Strengths and Weaknesses

The results in this paper are based on a very large dataset comprising more than 90,000 individual twins with information of high quality on zygosity and vital status. The mortality of individual twins is analyzed using an empirical approach and semiparametric models to conduct statistical inference, testing hypotheses and estimating hazard ratios, while accounting for the dependency within twin pairs and adjusting for birth cohorts by stratifying the baseline hazard function. 

Although the Danish Twin Registry was established in 1954, it includes birth cohorts from 1870 onwards. For twins in the earlier birth cohorts, before the register was established, zygosity of the twins was determined late in their lives, or even posthumously, leading to a relatively large proportion of twin pairs with UZ in the early cohorts. Due to the structure in church records, retrospective identification of opposite-sex twins was more difficult than identification of same-sex twins, which may explain why some of the earlier birth cohorts in the Danish Twin Registry do not capture as large a proportion of opposite-sex twins as same-sex twins. In the third cohort, birth cohorts 1930–1960, the presence of MZ and female twin individuals are lower than in the other cohorts which might lead to selection bias. To address potential confounding due to this bias in the survival analyses, all baseline hazards were stratified on cohorts and, in analyses combing both sexes, the baseline hazards were further stratified on sex. The criterion on survival until 1968 for all birth cohorts from 1930–1968 could introduce a healthy-survivor effect, although this effect is likely to be comparable for MZ and DZ twin individuals. 

In the 19th and first part of the 20th century, infant mortality was high overall and especially high for twins due to low birth weight. In the current paper as well as the paper by Sharrow and Anderson [9], twin pairs were included only if both had survived to age 6, respectively age 10, thus selecting only the hardiest twin pairs. This could lead to biased results on survival in favour of MZ twins over DZ twins because MZ twins have higher infant mortality. Another possible selection bias in the earlier cohorts may have been difficulties in classifying the zygosity of twins dying young since, in many cases, also their parents had died and they could not act as informants about zygosity. Thus, the high mortality seen in UZ twins in the early cohorts might be related to high mortality in the population in general rather than implying high early mortality in UZ twins.

## 5. Conclusions

The population-based and oldest twin cohort ever studied suggests that although direct comparisons of MZ and DZ twin individuals may indicate that they differ slightly in mortality, no substantial nor systematic difference in survival between MZ and DZ twin individuals is found when taking twins with unknown zygosity into consideration and accounting for the effects of birth- and age-cohorts, gender differences, and that twins are paired.

## Figures and Tables

**Table 1:** Population characteristics of individual twins, surviving to age 6, in the four cohorts, excluding UZ.

	All cohorts 1870–1990	Cohort 1 1870–1900	Cohort 2 1901–1930	Cohort 3 1931–1960	Cohort 4 1961–1990
Total, *n* (pairs)	96,338 (49,390)	9037 (4907)	15,645 (8060)	37,330 (19,114)	34,326 (17,309)
Age at FU ^a^, mean (SD)	58.5 (18.3)	69.0 (19.8)	74.0 (16.8)	65.3 (11.2)	41.2 (9.2)
Sex, *n* (%)					
Male	49,100 (51.0)	4496 (49.8)	7480 (47.8)	20,133 (53.9)	16,991 (49.5)
Female	47,238 (49.0)	4541 (50.3)	8165 (52.2)	17,197 (46.1)	17,335 (50.5)
Zygosity, *n* (%)					
MZ	23,888 (24.8)	2247 (24.9)	4613 (29.5)	7122 (19.1)	9906 (28.9)
SSDZ	39,728 (41.2)	4029 (44.6)	9156 (58.5)	14,440 (38.9)	12,103 (35.3)
OSDZ	32,722 (34.0)	2761 (30.6)	1876 (12.0)	15,768 (42.2)	12,317 (35.9)
Dead during FU ^a^, *n* (%)					
No/censored	62,459 (64.8)	208 (2.3)	959 (6.1)	27,806 (74.5)	33,486 (97.6)
Yes	33,879 (35.2)	8829 (97.7)	14,686 (93.9)	9524 (25.5)	840 (2.4)

^a^ FU is defined as time until death, censoring (emigration) or end of study period (1 October 2016). SD: standard deviation; MZ: monozygotic; SSDZ: same-sex dizygotic; OSDZ: opposite-sex dizygotic.

**Table 2:** Associations between mortality and zygosity in individual twins from all birth cohorts 1870–1990 and surviving to age 6; among individual twins at ages up to 50 years, between 50–75 years, and from 75 years.

	All Ages, *n* = 96,338 ^a^	Ages ≤50, *n* = 96,338 ^a^	Ages 51–75, *n* = 65,107 ^a^	Ages >75, *n* = 20,839 ^a^
**Both sexes**	HR (95%-CI)	HR (95%-CI)	HR (95%-CI)	HR (95%-CI)
Zygosity, *n* (%)				
MZ	Ref.	Ref.	Ref.	Ref.
SSDZ	1.09 (1.06, 1.12) ***	1.19 (1.11, 1.29) ***	1.11 (1.06, 1.17) ***	1.04 (1.00, 1.09) *
OSDZ	1.17 (1.13, 1.21) ***	1.53 (1.41, 1.66) ***	1.12 (1.06, 1.18) ***	1.09 (1.04, 1.15) ***
**Males**				
Zygosity, *n* (%)				
MZ	Ref.	Ref.	Ref.	Ref.
SSDZ	1.11 (1.07, 1.16) ***	1.23 (1.11, 1.36) ***	1.11 (1.04, 1.18) ***	1.07 (1.00, 1.13) *
OSDZ	1.16 (1.11, 1.21) ***	1.44 (1.30, 1.60) ***	1.10 (1.03, 1.18) **	1.11 (1.03, 1.19) **
**Females**				
Zygosity, *n* (%)				
MZ	Ref.	Ref.	Ref.	Ref.
SSDZ	1.07 (1.03, 1.12) **	1.20 (1.11, 1.30) ***	1.12 (1.07, 1.17) ***	1.04 (1.00, 1.08)
OSDZ	1.18 (1.13, 1.24) ***	1.52 (1.40, 1.64) ***	1.11 (1.06, 1.17) ***	1.09 (1.04, 1.15) ***

^a^ Baseline hazard stratified on cohort group (analyses on both sexes stratified on sex as well). HR: hazard ratio; CI: confidence interval; * *p* < 0.05, ** *p* < 0.01, *** *p* < 0.001.

**Table 3:** Population characteristics of individual twins, surviving to age 6, birth cohorts 1961–1990.

	All Subjects 1961–1990	Males 1961–1990	Females 1961–1990
Total, *n* (pairs)	39,504 (20,232)	19,964 (13,266)	19,540 (13,000)
Age at FU ^a^, mean (SD)	40.3 (9.6)	40.2 (9.5)	40.5 (9.6)
Sex, *n* (%)			
Male	19,964 (50.5)	19,964 (100.0)	-
Female	19,540 (49.5)	-	19,540 (100.0)
Zygosity, *n* (%)			
MZ	9906 (25.1)	4689 (23.5)	5217 (26.7)
SSDZ	12,103 (30.6)	6159 (30.9)	5944 (30.4)
OSDZ	12,317 (31.2)	6143 (30.8)	6174 (31.6)
UZ	5178 (13.1)	2973 (14.9)	2205 (11.3)
Dead during FU ^a^, *n* (%)			
No	38,519 (97.5)	19,334 (96.8)	19,185 (98.2)
Yes	985 (2.5)	630 (3.2)	355 (1.8)

^a^ FU is defined as time until death, censoring (emigration) or end of study period (1 October 2016).

**Table 4:** Associations between mortality and zygosity in individual twins, birth cohorts 1961–1990, surviving to age 6; among individual twins at ages up to 50 years, and from 50 years.

	All Ages, *n* = 39,504	Ages ≤50, *n* = 39,504	Ages >50, *n* = 8711
**Both sexes ^a^**	HR (95%-CI)	HR (95%-CI)	HR (95%-CI)
Zygosity			
MZ	Ref.	Ref.	Ref.
SSDZ	1.07 (0.89, 1.13)	1.10 (0.91, 1.34)	0.80 (0.46, 1.40)
OSDZ	1.21 (1.01, 1.45) *	1.23 (1.02, 1.48) *	1.05 (0.61, 1.79)
UZ	1.83 (1.47, 2.28) ***	1.89 (1.51, 2.37) ***	1.25 (0.49, 3.20)
**Males**			
Zygosity			
MZ	Ref.	Ref.	Ref.
SSDZ	1.01 (0.80, 1.28)	1.06 (0.83, 1.35)	0.63 (0.29, 1.37)
OSDZ	1.22 (0.97, 1.53)	1.23 (0.97, 1.55)	1.14 (0.56, 2.29)
UZ	1.86 (1.43, 2.42) ***	1.92 (1.46, 2.51) ***	1.26 (0.39, 4.11)
**Females**			
Zygosity			
MZ	Ref.	Ref.	Ref.
SSDZ	1.17 (0.88, 1.56)	1.19 (0.88, 1.62)	1.04 (0.46, 2.31)
OSDZ	1.19 (0.90, 1.58)	1.23 (0.91, 1.67)	1.14 (0.56, 2.28)
UZ	1.71 (1.13, 2.60) *	1.78 (1.15, 2.75) **	1.18 (0.26, 5.45)

^a^ Baseline hazard stratified on sex. * *p* < 0.05, ** *p* < 0.01, *** *p* < 0.001.

## Supplementary Materials

The following are available online at https://www.mdpi.com/2073-4425/10/2/166/s1, Figure S1: Kaplan-Meier survival curves of mortality for males in each of the four cohorts; Figure S2: Kaplan-Meier survival curves of mortality for females in each of the four cohorts; Figure S3: Kaplan-Meier survival curves for males and females in birth cohorts 1961–1990; Table S1: Age stratified associations between mortality and zygosity in individual twins, treating all UZ-twin pairs as MZ, birth cohorts 1961–1990; Table S2: Age stratified associations between mortality and zygosity in individual twins, randomising UZ-twin pairs 1:1 to either MZ or same-sex DZ, birth cohorts 1961–1990; Table S3: Age stratified associations between mortality and zygosity in individual twins, randomising UZ-twin pairs 1:2 to either MZ or same-sex DZ, birth cohorts 1961–1990.
