# Supplementary material for: Lifespans of Twins: Does Zygosity Matter?
Source: Genes (Basel). 2019 Feb 20;10(2):166. doi: 10.3390/genes10020166 (PMC6409769; doi:10.3390/genes10020166)
Supplement: Supplementary file 1 [file genes-10-00166-s001.pdf]

Supplementary material

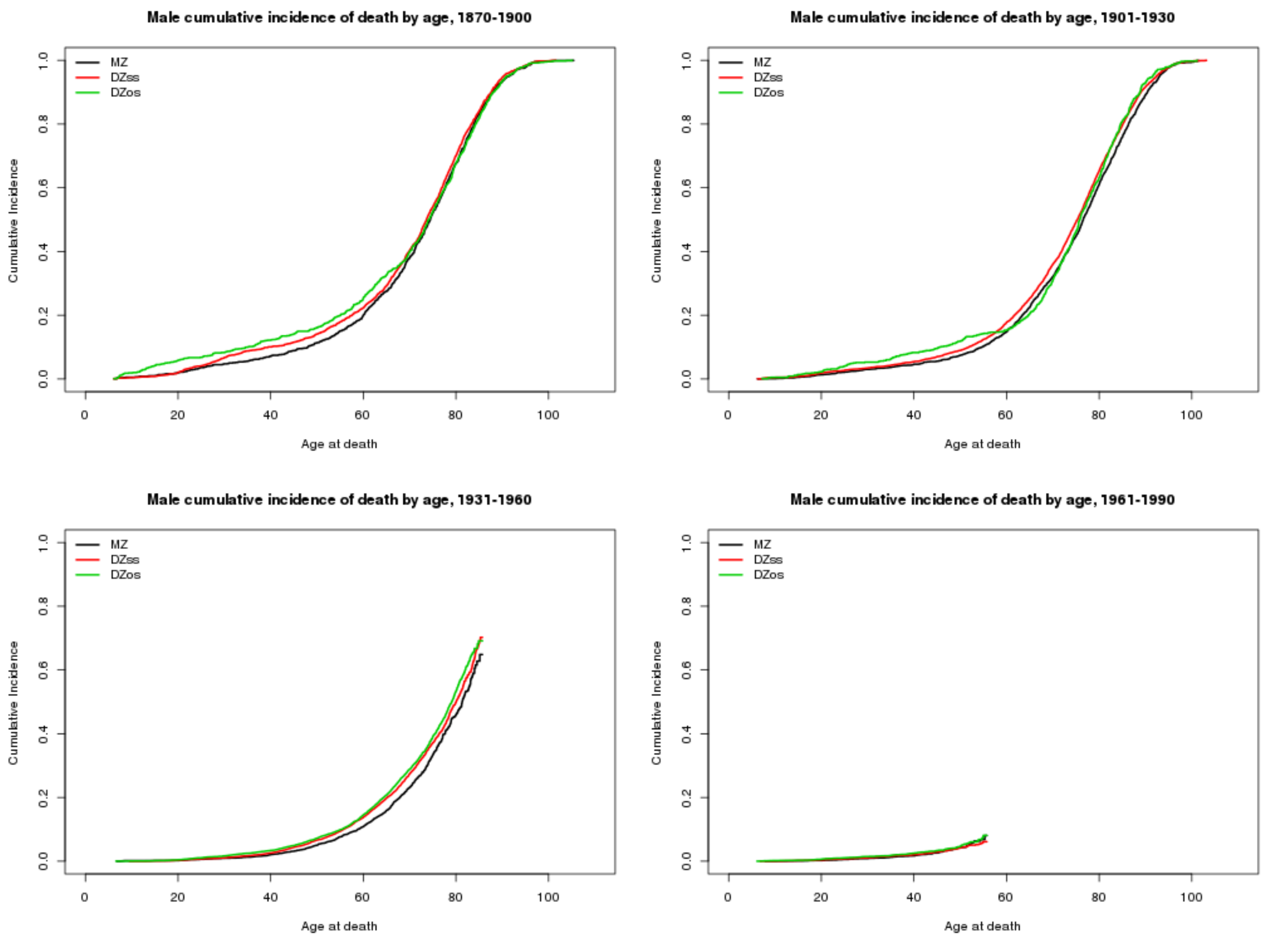

Figure S1. Cumulative incidence curves of mortality for males in each of the four cohorts

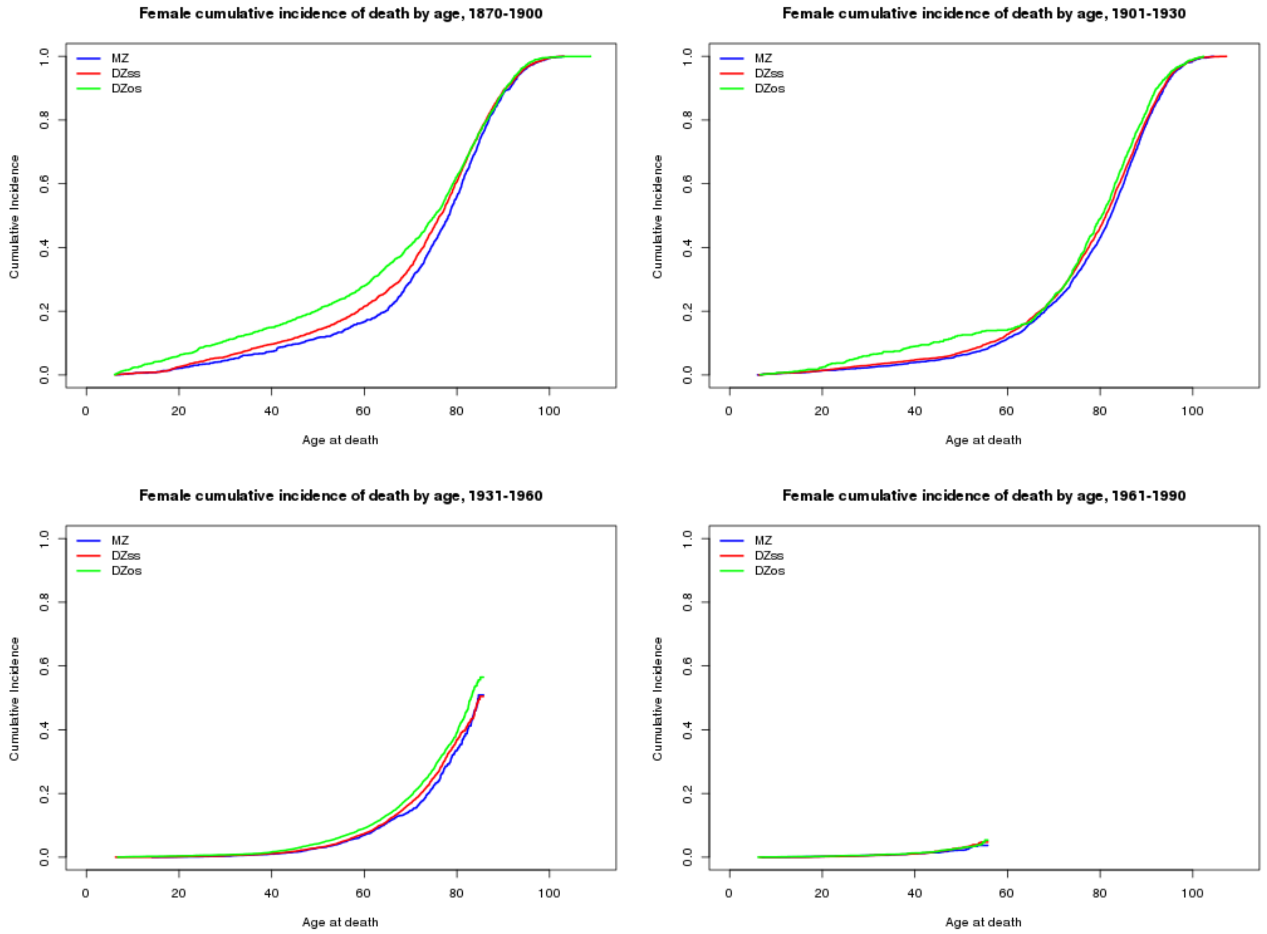

Figure S2. Cumulative incidence curves of mortality for females in each of the four cohorts

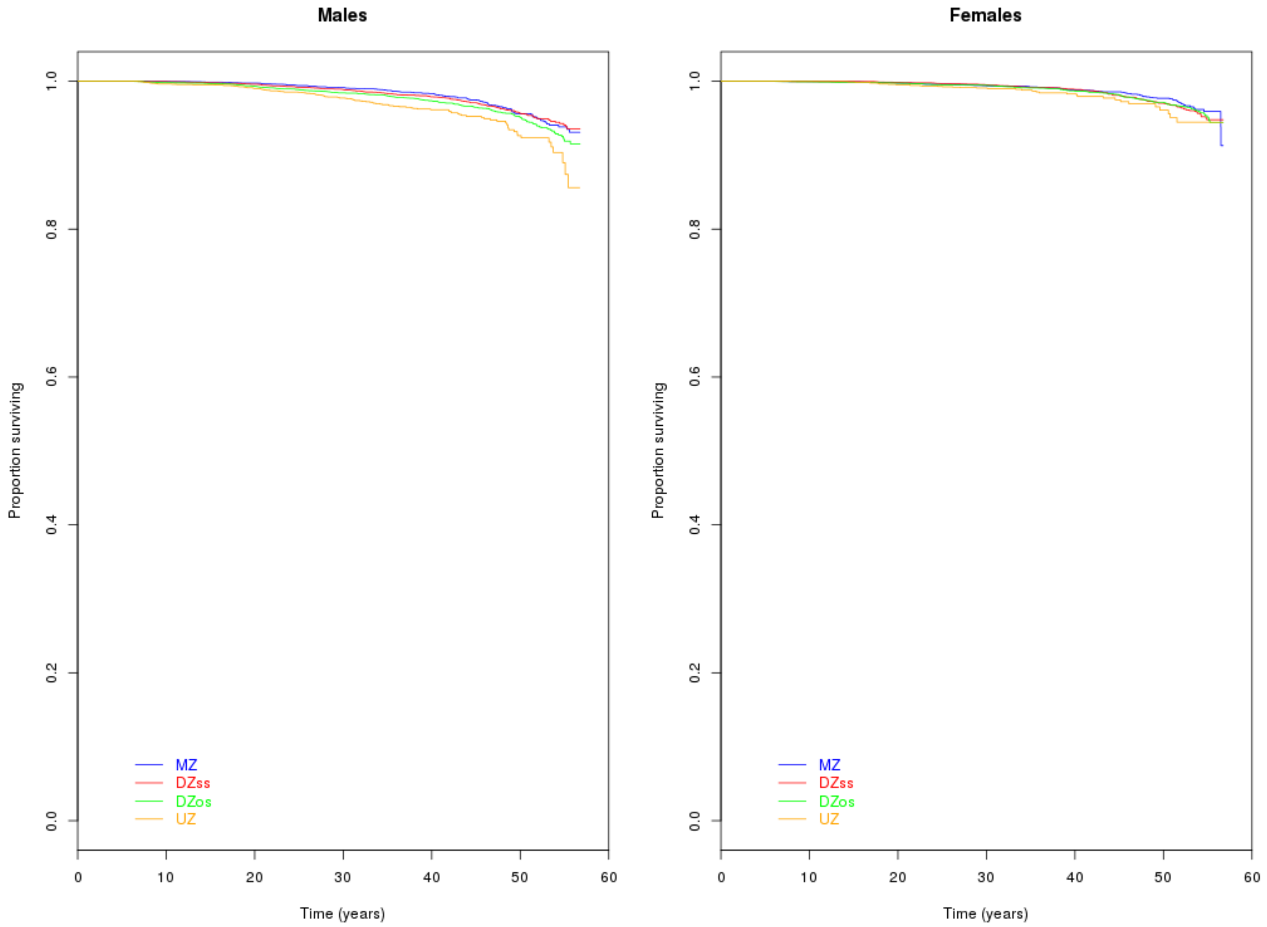

Figure S3. Kaplan-Meier survival curves for males and females in birth cohorts 1960–1990

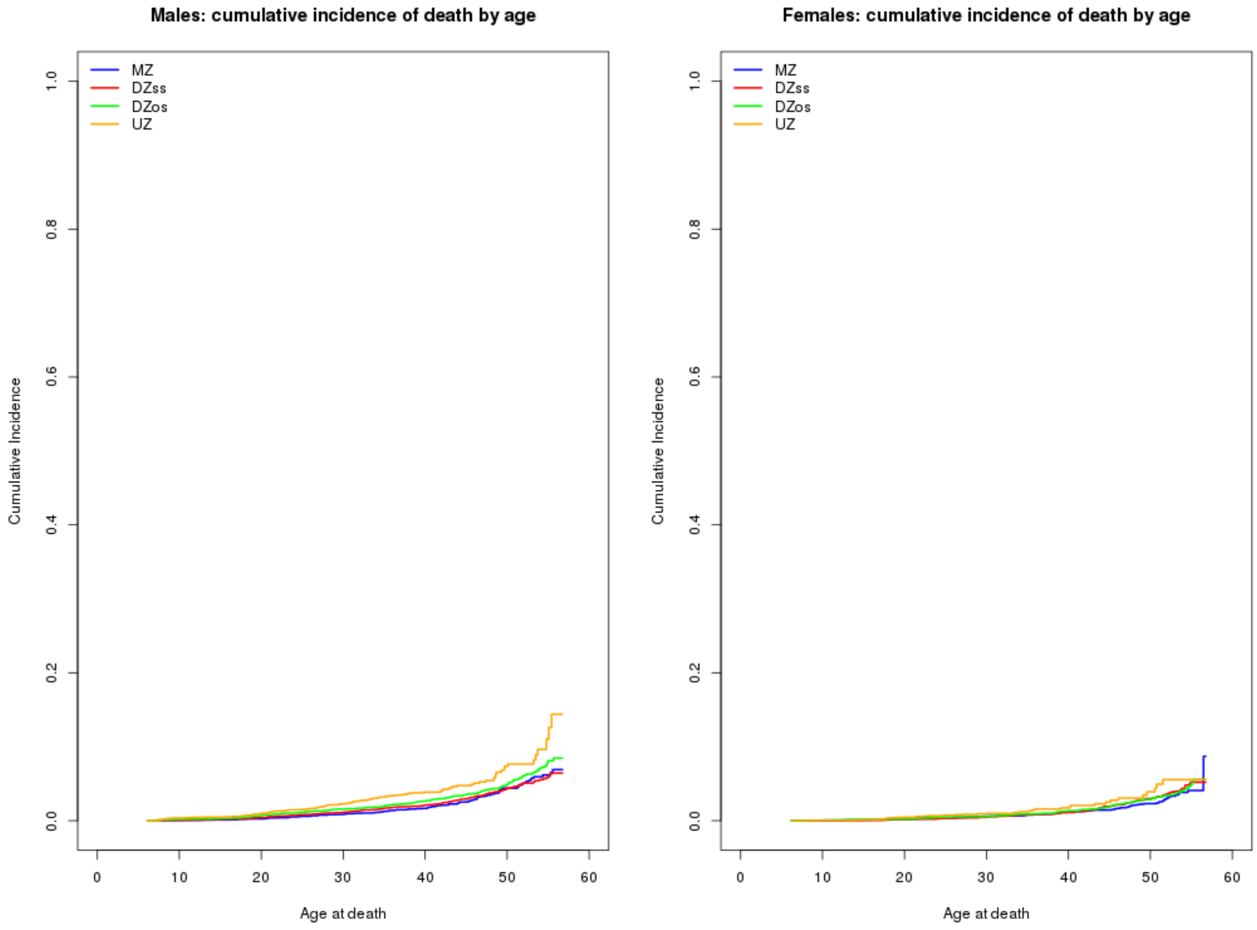

Figure S4. Cumulative incidence curves for males and females in birth cohorts 1960–1990

Table S1. Associations between mortality and zygosity in individual twins, treating all UZ-twin pairs as MZ, birth cohorts 1960–1990, surviving to age 6; among individual twins at ages up to 50 years, and from 50 years[a]

	All ages, n = 41,053	Ages ≤ 50, n = 41,053	Ages > 50, n = 10,134
**Both sexes**[b]	HR (95%-CI)	HR (95%-CI)	HR (95%-CI)
Zygosity, n (%)			
MZ	Ref.	Ref.	Ref.
SSDZ	0.90 (0.77, 1.04)	0.91 (0.77, 1.07)	0.82 (0.52, 1.29)
OSDZ	1.03 (0.89, 1.20)	1.02 (0.87, 1.19)	1.14 (0.75, 1.75)
**Males**			
Zygosity, n (%)			
MZ	Ref.	Ref.	Ref.
SSDZ	0.83 (0.68, 1.00)	0.84 (0.69, 1.03)	0.72 (0.38, 1.37)
OSDZ	1.03 (0.86, 1.23)	0.99 (0.82, 1.20)	1.33 (0.76, 2.33)
**Females**			
Zygosity, n (%)			
MZ	Ref.	Ref.	Ref.
SSDZ	1.03 (0.80, 1.31)	1.04 (0.80, 1.36)	0.93 (0.49, 1.77)
OSDZ	1.04 (0.82, 1.33)	1.07 (0.82, 1.39)	0.93 (0.49, 1.77)

[a]Composition of zygosities: 37.1% (MZ), 31.4 (SSDZ), 31.5 (OSDZ)
[b]Baseline hazard stratified on sex
Tests for interactions between zygosity and age-interval
All: p = 0.611, males: p = 0.288, females: p = 0.863

Table S2: Associations between mortality and zygosity in individual twins, randomising UZ-twin pairs 1:1 to either MZ or same-sex DZ, birth cohorts 1960-1990, surviving to age 6; among individual twins at ages up to 50 years, and from 50 years[a]

	All ages, n = 41,053	Ages ≤50, n = 41,053	Ages >50, n = 10,134
**Both sexes**[b]	HR (95%-CI)	HR (95%-CI)	HR (95%-CI)
Zygosity, n(%)			
MZ	Ref.	Ref.	Ref.
SSDZ	1.05 (0.90, 1.22)	1.09 (0.92, 1.28)	0.79 (0.50, 1.24)
OSDZ	1.12 (0.95, 1.30)	1.11 (0.94, 1.31)	1.11 (0.72, 1.71)
**Males**			
Zygosity, n(%)			
MZ	Ref.	Ref.	Ref.
SSDZ	1.01 (0.83, 1.23)	1.04 (0.85, 1.27)	0.76 (0.39, 1.43)
OSDZ	1.13 (0.93, 1.37)	1.10 (0.89, 1.34)	1.35 (0.75, 2.40)
**Females**			
Zygosity, n(%)			
MZ	Ref.	Ref.	Ref.
SSDZ	1.11 (0.87, 1.43)	1.18 (0.90, 1.54)	0.82 (0.43, 1.57)
OSDZ	1.09 (0.85, 1.41)	1.14 (0.87, 1.50)	0.86 (0.45, 1.65)

[a]Composition of zygosities: 31.4% (MZ), 37.2 (SSDZ), 31.4 (OSDZ)
[b]Baseline hazard stratified on sex
Tests for interactions between zygosity and age-interval
All: p = 0.593, males: p = 0.303, females: p = 0.811

Table S3: Associations between mortality and zygosity in individual twins, randomising UZ-twin pairs 1:2 to either MZ or same-sex DZ, birth cohorts 1960-1990, surviving to age 6; among individual twins at ages up to 50 years, and from 50 years[a]

	All ages, n = 41 053	Ages ≤50, n = 41 053	Ages >50, n = 10 134
**Both sexes**[b]	HR (95%-CI)	HR (95%-CI)	HR (95%-CI)
Zygosity, n (%)			
MZ	Ref.	Ref.	Ref.
SSDZ	1.13 (0.97, 1.32)	1.17 (0.99, 1.38)	0.85 (0.54, 1.34)
OSDZ	1.17 (1.00, 1.37)	1.17 (0.98, 1.38)	1.15 (0.74, 1.79)
**Males**			
Zygosity, n (%)			
MZ	Ref.	Ref.	Ref.
SSDZ	1.11 (0.91, 1.35)	1.14 (0.93, 1.40)	0.84 (0.44, 1.60)
OSDZ	1.19 (0.98, 1.46)	1.16 (0.94, 1.44)	1.41 (0.79, 2.54)
**Females**			
Zygosity, n (%)			
MZ	Ref.	Ref.	Ref.
SSDZ	1.15 (0.91, 1.51)	1.23 (0.94, 1.62)	0.86 (0.45, 1.65)
OSDZ	1.13 (0.87, 1.46)	1.18 (0.89, 1.56)	0.89 (0.46, 1.71)

[a]Composition of zygosities: 29.5% (MZ), 39.1 (SSDZ), 31.5 (OSDZ)
[b]Baseline hazard stratified on sex
Tests for interactions between zygosity and age-interval
All: $p = 0.351$, males: $p = 0.267$, females: $p = 0.539$
